# Packaging of disposable vaping products and e‐liquids in England, Canada and the United States: A content analysis

**DOI:** 10.1111/add.16611

**Published:** 2024-07-06

**Authors:** Matilda K. Nottage, Eve V. Taylor, Katherine A. East, Ann McNeill, James F. Thrasher, Jessica L. Reid, David Hammond, Erikas Simonavičius

**Affiliations:** ^1^ Addictions Department, Institute of Psychiatry, Psychology and Neuroscience King's College London London United Kingdom; ^2^ Department of Health Promotion, Education and Behavior, Arnold School of Public Health University of South Carolina Columbia South Carolina USA; ^3^ School of Public Health Sciences University of Waterloo Waterloo Ontario Canada

**Keywords:** branding, compliance, content analysis, disposable e‐cigarettes, e‐liquid, marketing, packaging, vaping

## Abstract

**Background and Aims:**

Vaping product packaging is varied and often features bright colours and novel designs, particularly among recently marketed disposable vapes. This study provides an overview of attributes found on the packaging of popular disposable vapes and e‐liquid bottles in England, Canada and the United States (US) and assesses compliance with local packaging regulations.

**Design:**

Content analysis.

**Setting:**

Brick‐and‐mortar and online shops in England (London), Canada (Ontario) and the US (New Hampshire and South Carolina).

**Cases:**

108 vaping products (including packaging) from 76 brands in a range of flavours and nicotine levels. Specifically, 48 disposable vapes (15 from England, 16 from Canada, 17 from the US) and 60 e‐liquid bottles (20 per country).

**Measurements:**

Textual and graphic branding and marketing elements, independently coded by two researchers and checked by a third.

**Findings:**

Compliance with local packaging regulations varied across countries. Health warnings were present on the packaging of all but one nicotine‐containing product, although 33% of disposables and 17% of e‐liquids featuring the warning did not adhere to formatting requirements. Leaflets were seldom included with e‐liquid bottles, even in England (45%) where mandatory, and omitted elsewhere. Labelling of nicotine type and batch numbers was inconsistent. Vaping product packaging featured claims relating to sensory perceptions (41%), most often flavours, and some (32%) featured youth‐appealing content. Common graphic elements included stylised brand fonts (80%), brand logos (54%), product representations on the external packaging (47%) and abstract graphic elements (64%). Colours on packaging, disposable vapes and e‐liquid bottles were associated with product flavour.

**Conclusions:**

In England, Canada and the United States, popular disposable vapes and e‐liquid bottles appear to have varying compliance with local packaging regulations and inconsistent labelling of nicotine and product characteristics. The use of colourful designs, evocative descriptors and appealing graphics to promote flavours underscores the need for comprehensive packaging regulations and enforcement.

## INTRODUCTION

The vaping product (VP) market is extensive, varied and rapidly evolving. Disposable devices, which are closed‐system products pre‐filled with e‐liquid, are increasingly popular in England, Canada and the United States (US), especially among youth [1, 2, 3]. Other VP types include refillable and rechargeable pod and tank devices, as well as e‐liquid bottles. The e‐liquid itself may be nicotine‐free or contain varying concentrations of freebase or salt‐based nicotine and come in flavours such as tobacco, fruit, sweets, menthol and other combinations [4, 5]. Vaping can support smoking cessation among adults who smoke [6], but the proliferation of diverse products and their growing popularity among youth complicate efforts to regulate packaging so it adequately reflects the risks and benefits of using VPs.

Packaging plays an important role in the perceptions and use of VPs. Among youth, exposure to VP marketing is associated with experimental VP use, reduced perceptions of harm and addictiveness of vaping and increased reported VP appeal [7, 8]. Furthermore, branded VP packs are more appealing to youth, but not adults, than standardised packs or ones without brand imagery [9, 10, 11]. This is in line with cigarette packaging, which has been studied extensively as a marketing medium [12]. Packaging characteristics, including branding, colours, shape, warnings and descriptors, influence the appeal of combustible cigarettes, particularly among youth [13, 14, 15, 16]. As a result, many countries, including England and Canada, have introduced regulations around combustible cigarette packaging aiming to limit product appeal, particularly to youth and people who do not smoke. Regulations limiting the use of tobacco cigarette packaging as a marketing medium can reduce smoking behaviour [17, 18].

VP packaging is already subject to regulations in several jurisdictions. For instance, the European Union Tobacco Products Directive and its derivative in the United Kingdom (UK), the Tobacco and Related Products Regulations (TRPR), require the inclusion of nicotine warnings on nicotine‐containing VPs, as well as an ingredients list, instructions for safe use, indication of nicotine contents and advice to keep the product away from children. The US Food and Drug Administration (FDA) require nicotine‐containing VPs to feature a warning about the addictiveness of nicotine on packages. In Canada, the Tobacco and Vaping Products Act (TVPA) also requires the presence of a nicotine warning on nicotine‐containing VPs, as well as warnings about hazards and toxicity, an ingredients list, indication of nicotine content and child‐resistant packaging. Noticing of nicotine health warning labels on VPs has been decreasing among youth across the three countries [19]; this may be because of ‘wearout’, a phenomenon known to affect cigarette warning labels, where the saliency of warning decreases over time as their novelty decreases [20].

In addition to mandating warnings and product information, some jurisdictions restrict promotional characteristics on VP packaging. In the UK, the TRPR prohibits ‘misleading’ packaging characteristics, such as claims about health or lifestyle benefits, claims relating to taste or smell (except flavourings), sustainability claims and financial promotions [21]. Canada restricts the promotion of youth‐appealing attributes on packaging, including dessert or confectionary flavours [22]. Any health‐related claims on VP packaging in the United States would require a modified risk tobacco product authorisation, which no VP has acquired yet [23]. Specific regulations for these three countries are described in Table 1. Marketing characteristics of VPs, such as branding, logos, or the use of colours and imagery, are not currently regulated in most countries. New Zealand regulates the use of toy or cartoon imagery on certain VPs, and a few jurisdictions, such as Israel and Finland, have implemented standardised VP packaging similar to standardised cigarette packaging [17, 25, 26]. Standardised packaging is being considered in the Netherlands, and the United Kingdom has recently announced new legislation that will introduce powers to regulate the display of VPs, packaging and flavours [17, 27].

**TABLE 1:** Codes and corresponding national/federal regulations in England, Canada and the United states at the time of data collection.

Category	Codes	Regulations		
		England	Canada	United States
Product characteristics: general	Country; product type (device or bottle); brand name; packaging elements (external packaging/box, leaflet); batch number	Leaflet required [TRPR 6.37(2)] Batch number required [TRPR 6.37(3)]	Leaflet only required if main display on pack is smaller than 15cm^2^ [VPLPR 17(2)]	No federal requirements
Product characteristics: flavour	Flavour name; flavour group (tobacco, menthol, fruit, sweet/dessert, mixed)	No requirements	Restricted flavour descriptors [TVPA IV.2(30.47–30.49)]	Since 2024: officially authorised VP products must be tobacco flavoured. At time of data collection: bans on non‐tobacco flavour in some states and cities
Product characteristics: nicotine	Presence of nicotine; nicotine concentration; indication of salt nicotine in the ingredients list	20 mg/mL upper limit [TRPR 6.36(4)]; List of all ingredients in the product in descending order by weight [TRPR 6.37(3)(a)]; Indication of the nicotine content of the product and the delivery per dose [TRPR 6.37(3)(b)]	20 mg/mL upper limit [NCVPR 4(1)]	No federal requirements
Product characteristics: e‐liquid volume	E‐liquid volume; number of ‘puffs’	Upper limits of 10 mL per e‐liquid bottle / 2 mL per disposable device [TRPR 6.36(2)]	100 mL	No federal requirements
Mandatory health warning	Presence of the warning on packaging; presence of the warning on the device and/or e‐liquid bottle; compliance with formatting requirements	Required on packaging of all nicotine‐containing products or e‐liquid bottles [TRPR 6.37(4)] ‘This product contains nicotine, which is a highly addictive substance’	Required on nicotine‐containing products [VPLPR 13] ‘WARNING: Nicotine is highly addictive. AVERTISSEMENT: La nicotine crée une forte dépendance’	Required on nicotine‐containing products ‘WARNING: This product contains nicotine. Nicotine is an addictive chemical’
Health‐related	Claims about: overall health; health benefits; smoking cessation; relative harm compared to smoking; natural or organic ingredients	Restricted [TRPR 6.38(3)(a‐b)]	Restricted [TVPA IV.2(30.43–30.44)]	Requires modified risk tobacco product authorisation, not yet issued to any VP in the US; Restricted claims around smoking cessation
Quality‐related	Claims about: product quality, origin of manufacturing and engineering (beyond factual mention of the manufacturer's address); recyclability; sustainability	Some restrictions [TRPR 6.38(3)(e)]	No requirements	No requirements
Financial	Price indications, promotions, claims about costs	Restricted [TRPR 6.38(4)]	Some restrictions [TVPA IV.2(30.6)]	Price promotions can be restricted at the state level
Social	Claims about social or romantic benefits; associations with personal identity; endorsements	Some restrictions [TRPR 6.38(3)(b)]	Some restrictions [TVPA IV.2(30.2–30.21)]	No requirements
Content appealing to youth	Packaging and marketing elements referring to fantasy, violence, humour or adventure^a^	No requirements	Some restrictions [TVPA IV.2 (30.1)]	No requirements
Sensory claims	Allusions to sensations associated with product use; flavour‐related imagery	Restricted [TRPR 6.38(3)(c‐d)]	Some restrictions [TVPA IV.2(30.46)]	No requirements
Graphic attributes: colour	Colours prominently used on packaging and products	No requirements	No requirements	No requirements
Graphic attributes: shape	Shape of packaging or products	No requirements	No requirements	No requirements
Graphic attributes: brand names and logos	Brand names; stylised fonts; logos	No requirements	No requirements	No requirements
Graphic attributes: graphics and imagery	Representation of the product on the external packaging; flavour‐related imagery; abstract or figurative graphic elements on packaging or products	No requirements	No requirements	No requirements
Digital elements	QR codes; reference to websites or social media	No requirements	No requirements	No requirements

Abbreviations: NCVPR, Nicotine Concentration in Vaping Products Regulations; QR, quick response; TRPR, Tobacco and Related Products Regulations; TVPA, Tobacco and Vaping Products Act; VP, vaping products; VPLPR, Vaping Products Labelling and Packaging Regulations.

^a^ Adapted from the index developed by Padon et al. [24].

Regulating VP packaging is complex because of the diversity of devices and products [28, 29]. Ideally, packaging regulations should convey the potential harm of VPs, as well as the lower relative risk compared to smoking [30, 31, 32, 33]. Indeed, certain standardised packaging designs have been associated with increased misperceptions of the relative harm of vaping compared to smoking [11]. Therefore, existing VP packaging features should be studied and considered to inform the development of specific packaging regulations, with consideration for compliance issues. It is important to balance discouraging VP uptake by youth and people who do not smoke without discouraging people who smoke from using VPs as a cessation aid or harm‐reducing alternative. To our knowledge, no recent published academic research has comprehensively analysed the contents of VP packaging and their compliance with packaging regulations.

The present study aimed to provide an overview of branding, marketing and labelling attributes (textual and graphic) on popular disposable VPs and e‐liquids, considering both the external packaging and the products themselves. We investigated popular products sold in England, Canada and the United States, and assessed compliance with local regulations; VPs are widely available across these countries, yet their packaging regulations differ [30]. This study focused primarily on disposable VPs because they are increasingly popular among youth and young adults in the United States, Canada and England [1, 2, 3, 34]. The inclusion of both disposable devices and e‐liquid bottles allows an exploration of packaging characteristics relating to flavours and nicotine.

## METHODS

This study comprises a content analysis of disposable devices and e‐liquids and their packaging, across three countries.

### Sample

The protocol for this project, including details of the sampling strategy, was published on the Open Science Framework [35]. Briefly, a convenience sample of popular disposable VPs and e‐liquid bottles, with and without nicotine, were obtained from online and brick‐and‐mortar retailers in England (London), Canada (Waterloo, Ontario) and the United States (brick‐and‐mortar in Nashua, New Hampshire, and online delivered to Columbia, South Carolina) between February 2022 and January 2023. We sampled popular VPs using data from four nationally representative online surveys, including adults and youth. In each country, we selected the most used flavour categories, product types and the top three brands of disposable devices [36, 37, 38]. Each product was independently double‐coded. Discrepancies were resolved by a third researcher through inspection of the product and, when needed, discussion with the original coders.

#### Deviation from the pre‐registered sampling frame

The initial sample selection methodology [35] was amended during product purchases because of product availability and shifting market trends. Specifically, several of the disposable device brands initially selected for Canada and the United States were unavailable on most retail websites; these were replaced with a selection of brands that appeared on top of the ‘best‐selling’ rankings across multiple online retailers. The retail websites used were the top five results on Google for each of the searches ‘vape shop US’ and ‘vape shop Canada’. Whereas we planned to sample two products per brand from the most popular flavour category, we sampled one instead, because of similarities between products and packaging of the same brand. Finally, we had originally intended to sample pod devices, but these were excluded because of difficulty obtaining these online in Canada and the United States, particularly products for flavours other than tobacco or menthol, possibly reflecting the effect of regulatory restrictions.

### Codebook

A codebook was iteratively developed, initially based on TRPR requirements for VPs in England, then augmented as appropriate when assessing products from Canada and the United States (Table S1). Details of the codebook development process are described in the study protocol [35]. Codes used in the present study are described in Table 1. Several codes were accompanied by an open text field to provide and specifications about that code (Table S1). Both graphic and textual data were considered when coding, including explicit attributes (e.g. nicotine concentration, presence of the health warning) and implicit ones (e.g. sensory claims, content appealing to youth). Coder agreement was strong for explicit codes; the third coder was required to settle inconsistencies in several instances for implicit codes.

### Analysis

Data was analysed using Microsoft Excel. We report product characteristics and how frequently each code was identified on disposable devices or e‐liquid bottles and their packaging. Although we report the inclusion of other packaging elements, such as leaflets and peel‐off labels, their contents were not coded. Some codes apply universally, although others pertain specifically to disposables or e‐liquid bottles and are reported separately. Additional data are narratively presented. Primary findings concern marketing and branding of VPs and compliance with respective country regulations, as described in Table 1. For the analysis of product and packaging colours used in relation to flavours, colours were categorised as either: black, white, neutral (grey, metallic, brown), warm (red, orange, yellow, pink) or cool (green, blue, purple). If a product or its packaging prominently featured two colours from different categories (e.g. half white and half black), both were coded. Findings are reported by VP type (disposable or e‐liquid) and by country.

## RESULTS

The final sample consisted of 108 products from 76 individual brands: 48 disposable devices (15 from England, 16 from Canada, 17 from the United States) and 60 e‐liquid bottles (20 from each country). A description of the total sample, including product characteristics, is provided in Table 2, alongside results for the coded marketing and branding attributes. The full product list can be found in Table S2.

**TABLE 2:** Characteristics of the sampled disposable and e‐liquid vaping products (*n* = 108).

	England		Canada		United States	
	Disposables (*n* = 15)	E‐liquid bottles (*n* = 20)	Disposables (*n* = 16)	E‐liquid bottles (*n* = 20)	Disposables (*n* = 17)	E‐liquid bottles (*n* = 20)
	*n* (%)	*n* (%)	*n* (%)	*n* (%)	*n* (%)	*n* (%)
Packaging						
Included: external packaging/box	15 (100)	13 (65)	16 (100)	0	17 (100)	14 (70)
Included: leaflet	14 (93)	9 (45)	13 (81)	0	4 (24)	0
Included: batch number	13 (87)	19 (95)	13 (81)	19 (95)	11 (65)	17 (85)
Nicotine concentration						
0 mg/mL	0	9 (45)^a^	0	7 (35)	0	7 (35)
1–10 mg/mL	0	4 (20)	0	5 (25)	0	5 (25)
11–20 mg/mL	15 (100)	7 (35)	16 (100)	8 (40)	0	1 (5)
21–30 mg/mL	0	0	0	0	0	2 (10)
31–40 mg/mL	0	0	0	0	0	2 (10)
41–50 mg/mL	0	0	0	0	16 (94)	3 (15)
>50 mg/mL	0	0	0	0	1 (6)	0
E‐liquid volume						
Unspecified	1 (7)	0	0	0	0	0
<2 mL	1 (7)	0	0	0	1 (6)	0
2 mL	13 (87)	0	3 (19)	0	1 (6)	0
3–9 mL	0	0	9 (56)	0	8 (47)	0
10 mL	0	15 (75)	3 (19)	0	0	0
11–50 mL	0	4 (20)	1 (6)	7 (35)	7 (41)	8 (40)
51–100 mL	0	1 (5)	0	13 (65)	0	12 (60)
Health warnings						
Included on nicotine‐containing products	15 (100)	11/11^b^ (100)	16 (100)	12/13^b^ (92)	17 (100)	13/13^b^ (100)
Format: compliant	11 (73)	10/11^b^ (91)	15 (94)	10/13^b^ (77)	6 (35)	7/13^b^ (54)
Format: too small	3 (20)	1/11^b^ (9)	1 (6)	2/13^b^ (15)	7 (41)	4/13^b^ (31)
Format: non‐standard colours/font	1 (7)	0	0	0	1 (6)	0
Format: non‐standard text	0	0	0	0	6 (35)	2/13^b^ (15)
Warning place: packaging only	15 (100)	6/11^b^ (55)	16 (100)	N/A^c^	17 (100)	1/13^b^ (8)
Warning place: product only	0	2/11^b^ (18)	0	12/13^b^ (92)	0	2/13^b^ (15)
Warning place: packaging and product	0	3/11^b^ (27)	0	N/A^c^	0	9/13^b^ (69)
Flavours						
Tobacco	2 (13)	3 (15)	4 (25)	3 (15)	2 (12)	4 (20)
Menthol	3 (20)	4 (20)	4 (25)	4 (20)	3 (18)	4 (20)
Fruit	4 (27)	7 (35)	5 (31)	9 (45)	4 (24)	7 (35)
Sweet/Dessert	1 (7)	1 (5)	3 (19)	2 (10)	4 (24)	3 (15)
Mixed^d^	5 (33)	5 (25)	0	2 (10)	4 (24)	2 (10)
Health‐related claims						
Overall health	0	0	0	0	0	3 (15)
Health benefits	0	0	0	0	0	0
Smoking cessation	0	0	0	0	2 (12)	3 (15)
Relative harm compared to smoking	0	0	0	0	0	0
Natural ingredients	13 (87)	4 (20)	1 (6)	1 (5)	10 (59)	11 (55)
Quality‐related claims						
Quality markings	15 (100)	0	9 (56)	10 (50)	10 (59)	0
Quality claims	0	8 (40)	0	2 (10)	5 (29)	6 (30)
Origin claims	15 (100)	12 (60)	11 (69)	1 (5)	17 (100)	8 (40)
Recyclability (factual)	15 (100)	18 (90)	16 (100)	20 (100)	12 (71)	20 (100)
Sustainability claims	0	1 (5)	0	0	0	0
Financial claims						
Price indications	0	1 (5)	0	0	0	0
Promotions	0	1 (5)	0	0	0	0
Claims about costs	0	0	0	0	0	0
Social claims						
Social/romantic benefits	0	0	0	0	0	3 (15)
Personal identity	0	2 (10)	0	0	1 (6)	2 (10)
Endorsements	0	0	0	0	0	0
Content appealing to youth^e^	2 (13)	7 (35)	2 (13)	10 (50)	5 (29)	9 (45)
Sensory claims	2 (13)	12 (60)	5 (31)	9 (45)	5 (29)	11 (55)
Product shape						
Cylindrical (device)	9 (60)	N/A	12 (75)	N/A	7 (41)	N/A
Rectangular (device)	3 (20)	N/A	2 (13)	N/A	6 (35)	N/A
Mixed shape (device)	3 (20)	N/A	2 (13)	N/A	4 (24)	N/A
Common (bottle)	N/A	18 (90)	N/A	19 (95)	N/A	20 (100)
Uncommon (bottle)	N/A	2 (10)	N/A	1 (5)	N/A	0
Graphics and imagery						
Stylised brand font	8 (53)	17 (85)	10 (63)	19 (95)	16 (94)	16 (80)
Separate brand logo	10 (67)	13 (65)	9 (56)	10 (50)	3 (18)	13 (65)
Product image on external packaging	14 (93)	1/13 (8)	8 (50)	N/A^c^	11 (65)	1/14 (7)
Flavour‐related images	1 (7)	3 (15)	2 (13)	8 (40)	5 (29)	6 (30)
Graphic elements: abstract^f^	11 (73)	15 (75)	14 (88)	5 (25)	14 (82)	10 (50)
Graphic elements: figurative^f^	2 (13)	7 (35)	4 (25)	10 (50)	3 (18)	8 (40)
Digital elements						
Reference to brand's website	15 (100)	17 (85)	8 (50)	0	14 (82)	16 (80)
QR codes	10 (67)	2 (10)	5 (31)	0	13 (76)	5 (25)
Reference to social media	4 (27)	9 (45)	1 (6)	3 (15)	3 (18)	9 (45)

Abbreviation: QR, quick response.

^a^ Of these, four were ‘shortfills’: nicotine‐free e‐liquid bottles larger than 10 mL, which can be supplemented with nicotine ‘shots’.

^b^ Denominators reflect the number of nicotine‐containing e‐liquids.

^c^ None of the sampled e‐liquid products in Canada featured external packaging.

^d^ Across countries, flavour combinations included fruit and sweet/dessert (*n* = 9; e.g. ‘strawberry cheesecake’), tobacco and sweet/dessert (*n* = 5; e.g. ‘caramel tobacco’) and fruit and menthol (*n* = 4, e.g. ‘sour apple ice’).

^e^ Adapted from the index developed by Padon *et al*. [36].

^f^ Examples of abstract and figurative graphic elements can be found in Figure 2.

### Product, packaging and labelling characteristics

Product, packaging and labelling characteristics are shown in Table 2. All disposables were sold with external packaging, either cardboard (92%) or clear plastic (8%, United States only) containers. E‐liquid bottles were often packaged in cardboard boxes in England (65%) and the United States (70%), whereas none from the Canadian sample had external packaging. Some e‐liquids featured loose tags with ingredients lists (10% in Canada) or peel‐off labels with information underneath (25% in England and 10% in the United States).

Overall, 85% of VPs included batch numbers. VPs in the United States featured high concentrations of nicotine (up to 55 mg/mL), in contrast with England and Canada where, in compliance with regulations, no VPs indicated exceeding 20 mg/mL of nicotine. In England, nicotine‐containing VPs were compliant with e‐liquid volume restrictions. Nicotine‐free ‘short‐fill’ e‐liquid bottles were larger; one was accompanied by a 10 mL nicotine ‘shot’ bottle. Without federal volume restrictions in Canada and the United States, nicotine‐containing disposables indicated up to 13 mL (Canada) and 15 mL (US), and nicotine‐containing e‐liquid bottles up to 60 mL (Canada) and 100 mL (US).

Mentions of nicotine salts on the product or packaging (including ingredients lists) were identified on 33% of disposables in England, 75% of disposables in Canada and 29% of disposables in the United States. Approximately half of the nicotine‐containing e‐liquid bottles across the three countries (55% in England; 46% in Canada; 54% in the United States) included mentions of nicotine salts. In England, 80% of disposables claimed a capacity of 600 puffs, with a range of 575 to 1200 (one unspecified). Claimed puff capacity ranged from 500 to 5000 in Canada, with three disposables unspecified, and from 500 to 7000 in the United States, with one disposable unspecified.

### Health warnings

All disposables' packaging featured a health warning (Table 2), however, 33% did not correspond to the required formatting—the warning was too small, non‐standard in colours and fonts or featured non‐standard text (the latter only in the US sample, specifically the inclusion of additional phrases: ‘This product contains *tobacco‐free* nicotine’ and ‘*This product is not derived from tobacco*’). The health warning was included on all nicotine‐containing e‐liquids in England and the United States, and all but one in Canada; 25% of those did not meet formatting requirements. Among the 19 nicotine‐containing e‐liquids with external packaging (England and United States), 63% included health warnings on both packaging and product and 37% on the external packaging only. Although the warning is not required on nicotine‐free e‐liquid bottles (n = 23), 9% of products featured it.

### Heath‐related claims

The only health‐related claims identified across all countries were variations of references to ‘natural and artificial flavours’ found in the ingredient lists of 37% of VPs. In the United States, some claims related to smoking cessation (e.g. ‘This product is not a smoking cessation product’, ‘Thank you for not smoking’) and other health‐related claims (e.g. ‘This product is not intended to diagnose, treat or cure illness’) were identified (Table 2).

### Quality‐related claims

Quality markings (e.g. the European ‘CE’ marking) were identified on most disposables, but fewer e‐liquids, as shown in Table 2. Textual claims about product quality included claims such as ‘Premium quality’, ‘Trust what you inhale’ and ‘Luxury at its finest’. Claims about product origin (where it was manufactured or engineered, beyond a factual mention of the manufacturer's address) were identified on all disposables in England and the United States and on two‐thirds of disposables in Canada, but rarely on e‐liquid bottles (Table 2). Forty‐six percent of the 108 VPs featured text‐only claims (e.g. ‘Manufactured in Great Britain’, ‘Bottled in sunny Los Angeles, California’, ‘Made in China’), 12% featured both text and images (e.g. country flags), and 1% only an image. Ninety‐four percent of VPs featured recycling logos and/or disposal instructions, yet only one e‐liquid bottle included a claim concerning sustainability (the ‘Programme for the Endorsement of Forest Certification’).

### Financial claims

Only two instances of financial claims or promotions were identified, both in England (Table 2). One e‐liquid had a price printed on its external packaging; another featured a sticker covering a quick‐response (QR) code that stated: ‘Scan & win $250,000 in cash prizes’.

### Social claims

Social claims were rare across the sampled VPs (Table 2). Examples of textual claims relating to personal identity included ‘Thanks dude’ and ‘#teamvv’, and brand names ‘Keep it 100’ and ‘American Patriots’. Claims about social benefits included ‘Connect with us: @ [brand name]’, ‘Follow us on social’ and ‘Drip with us’.

### Content appealing to youth

Content appealing to youth was identified on 32% of all VPs (18% of disposables and 43% of e‐liquids) (Table 2). Of these, 23% featured fantasy themes through text and images (e.g. ‘monster’, ‘unicorn’, ‘magic’, illustrations of vampires and krakens), and 26% featured the theme of violence (e.g. ‘riot’, ‘vice’, ‘cartel’, illustrations of fists, skulls and comic‐book style explosions). Other content appealing to youth included product name and illustrations alluding to surfing, the use of street‐art style imagery and colourful illustrations of items reminiscent of childhood (e.g. a carousel, a bowl of breakfast cereal).

### Sensory claims

Claims about sensory perceptions were identified on 41% of VPs in this study, on 53% of e‐liquids and 25% of disposables and they usually concerned product flavours. Some sensory claims were communicated through imagery (39%), such as flavour‐related images of fruits, mint or tobacco leaves, or sensation‐related visual elements, like liquid droplets or clouds of smoke. Other sensory claims were textual (36%), ranging from evocative names (e.g. ‘icy mint’) to intricate descriptions (e.g. ‘This distinctive full‐bodied tobacco expertly blends mild notes of creamy Madagascan vanilla with luxurious British toffee’). Of all VPs featuring these claims, 25% used both text and images.

### Graphic attributes

#### Colour

**FIGURE 1:**
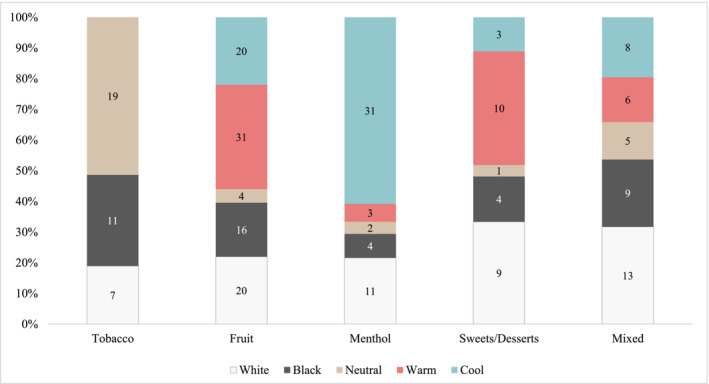
Prominently featured colours across vaping products and their packaging, by flavour group. *Note*. Up to two prominent colours were coded per product and per packaging.

Prominent colours on VPs and their packaging showed a clear pattern according to the flavour of the product, as shown in Figure 1 (data shown in Table S3), with mostly neutral colours reflecting tobacco, warm colours reflecting fruit and sweet/dessert and cool colours reflecting menthol flavours. For both disposables and e‐liquids, similar colours were used to represent flavour categories. One exception was sweets/desserts flavours, which were mostly conveyed by white colours for e‐liquids and by warm colours for disposables (Figure S1).

#### Shape

Across the three countries, most disposables (58%) had a cylindrical shape and all but three e‐liquids (95%) had a common cylindrical/round shape (Table 2). The uncommonly shaped bottles, two from the English sample and one from the Canadian sample, had unusual lid shapes (e.g. a tall and pointy bullet‐shaped lid).

#### Branding and logos

Branding, including the brand name and/or brand logo, was incorporated on all VPs and, where present, their packaging. Overall, 80% of VPs featured a brand name in a stylised font (71% of disposables and 87% of e‐liquids), of which 50% were heavily stylised. Two‐thirds of disposables in England, 56% in Canada and 18% in the United States featured a brand logo distinct from the brand name, of which five included figurative elements (e.g. leaves, bubbles and crown) in England, one in Canada and none in the United States (Table 2).

#### Other graphics

**FIGURE 2:**
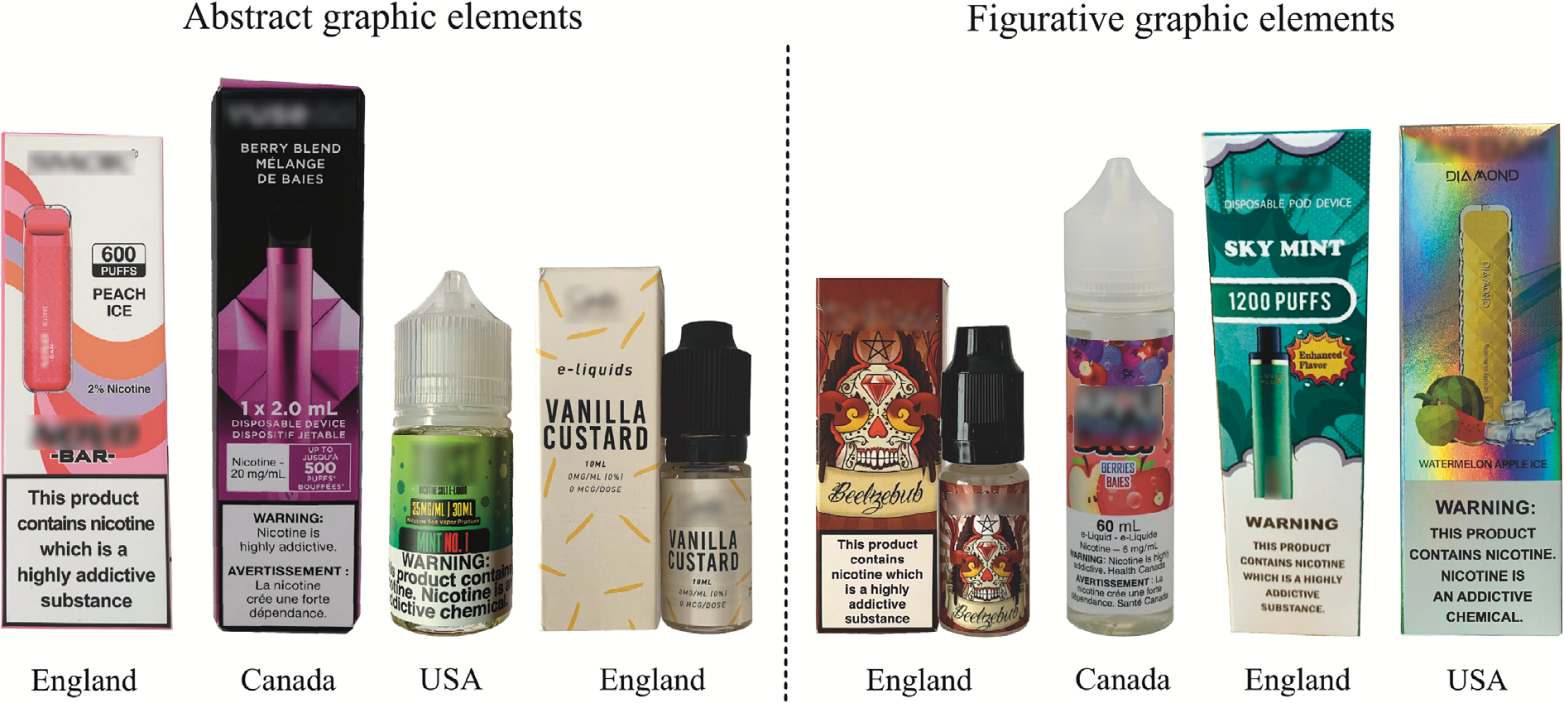
Example products featuring abstract and figurative graphic elements.

Most disposables (69%) featured an image representing the device on the external packaging, compared to only 7% of e‐liquids with external packaging (Table 2). Across countries, flavour‐related images appeared on the external packaging of 17% of disposables and 28% of e‐liquids, and most disposables (88%) and e‐liquids (82%) featured some other graphic elements (Table 2). Of these graphic elements, abstract ones were identified on 81% of disposables and 50% of e‐liquids, such as geometric patterns, abstract shapes, colour gradients, iridescence or textures such as embossing or contrasting glossy and matte areas. Figurative graphic elements, such as clouds, plant leaves, lightning bolts or cartoon‐style characters, were identified on 19% of disposables and 42% of e‐liquids. Examples of abstract and figurative elements are in Figure 2.

### Digital elements

Most disposables (77%) referenced the brand's website (Table 2). QR codes were identified on the external packaging of 58% of disposables, all directing to the brand's website. Several QR codes on disposables were accompanied by a ‘scratch‐to‐reveal’ element (27%; three in England and 10 in the United States) or mentioned authenticity checks (27%; seven in England, one in Canada, five in the United States). References to social media were identified on the packaging of 17% of disposables across countries. Most e‐liquids featured a reference to the brand's website in England and the United States, but none in Canada (Table 2). Overall, QR codes were identified on 12% of e‐liquids and references to social media on 35% of e‐liquids (Table 2). Social media platforms were referred to by name, icon or ‘hashtags’ relating to the brand, with a few explicit calls to action, like ‘Find the Yeti on socials!’.

## DISCUSSION

This study highlights the variety of marketing and branding attributes commonly found on popular disposable devices and e‐liquid bottles purchased in three countries with varying regulations—England, Canada and the United States. Non‐compliance was common among both disposables and e‐liquids, including non‐standard health warnings. Inconsistent nicotine labelling on packaging and sensory transference of flavours through graphic elements, colours and claims relating to sensory perceptions were the most striking marketing attributes. We discuss these findings with a particular focus on the increasingly popular disposable devices [1, 2, 3].

Nearly one‐third of the sampled VPs included marketing elements with content appealing to youth, and these were particularly common on disposables. Given the increasing concern about vaping among young people who never smoked [3], regulations for VP packaging elements that may appeal to youth should be better defined, constrained and enforced. For instance, New Zealand implemented legislation in March 2024 specifically prohibiting the use of toy or cartoon imagery on reusable VPs [26]. Moreover, the United Kingdom is currently drafting policy to bring powers to regulate the appearance of vape packaging [27].

Claims about sensory perceptions were identified on almost half of the overall sample. These sensory claims, together with graphic attributes of VP packaging, primarily emphasised the products' appeal by highlighting characteristics relating to flavours. There are widespread concerns that flavours and taste are key drivers of youth vaping [39]. We found that claims about sensory perceptions were particularly common on e‐liquid bottles, which may be because of flavour being one of the main distinguishing features of e‐liquid products, whereas disposable VPs could be marketed based on various characteristics, including design, branding or engineering features.

In addition to sensory claims, we identified the use of colours in the marketing of VPs to portray different flavours, consistent with sensation transference [40]. This was a common strategy of the tobacco industry for cigarettes with, for example, green portraying menthol flavours and lighter colours signifying cigarettes that purported to have lower nicotine and tar exposures [40]. Prominently featured colours across VPs were often used alongside corresponding flavour groups, e.g. neutral colours were mostly used to depict tobacco flavours, cool colours for menthol flavours and warm colours for fruit and sweet/dessert flavours. Flavour‐related images were relatively rare on the packaging of disposables and e‐liquids, which suggests that flavours were more likely conveyed through colours and flavour descriptors on packaging rather than through images. Nevertheless, various graphic elements, stylised brand names and logos were commonly featured elements used to promote the sampled VPs. Additionally, digital references via QR codes to brand websites were common marketing elements on VPs from England and the United States, but not Canada.

External packaging is not mandated in any of the three countries, yet leaflets appeared to be included more commonly where external packaging was present. Leaflets are required on all VPs in England by the TRPR and in Canada by the TVPA when the front of the pack is smaller than 15 cm^2^, but are not federally required in the United States. In line with these regulations, we identified leaflets included with most disposables in England and Canada, but only one‐quarter of disposables in the United States. All disposables were sold in external packaging, typically cardboard boxes. In contrast, e‐liquid bottles were often sold without external packaging, and leaflets were rarely included, even in England where it is required. External packaging and leaflets allow the inclusion of information important to the safe use and storage of VPs. Their absence may contribute to the risk of harmful exposure among children. In England, nearly half (48.8%) of the acute poisoning episodes caused by tobacco and nicotine (not necessarily vaping) recorded by Hospital Episode Statistics from 2015 to 2021 involved children under the age of 4 years, often including children who accidentally swallow e‐liquids [31]. Mandating both external packaging and leaflets may increase compliance and reduce the risks to children.

Almost all VPs displayed the mandatory health warnings, however, a considerable minority of these warnings did not adhere to the required formatting. Specifically, most of the products with non‐compliant health warnings were from the United States, frequently featuring non‐standard text indicating that the nicotine in the product was not derived from tobacco. These cases were possibly related to earlier unsuccessful attempts to circumvent the FDA's tobacco products regulations by promoting synthetic nicotine [39], which might increase youth intentions to purchase e‐cigarettes [41]. Additionally, inconsistencies in product labelling were prevalent. Notably, nicotine salts were not routinely indicated on disposables, including the ingredients list, despite their common presence in such products [42]. Some products across the three countries were also missing batch numbers. Indication of nicotine salts as ingredients and batch numbers are mandatory in England, but not in Canada or the United States. Therefore, similar inconsistencies in the VPs labelling across the three countries suggest that VP producers do not comply with all requirements equally.

The stated number of ‘puffs’ relative to e‐liquid volume varied widely among VPs from Canada and the United States, but was more consistent in the English sample, where all disposable devices contained 2 mL of e‐liquid, per TRPR requirements. In Canada, devices containing 10 mL featured claims of 3000 to 5000 puffs, whereas in the US, devices containing 15 mL featured claims of 5000 to 7000 puffs. Although the claimed range of ‘puffs’ might depend on device characteristics, people who vape prefer longer‐lasting disposables [43], and the prominent display of this information on packaging suggests it is an important, yet unregulated, marketing element for disposable VPs. Similar inconsistencies, including varied ways of reporting nicotine dose, were highlighted in Moodie and colleagues' [44] report on VP packaging in the United Kingdom and might influence youth vaping. Research has found that youth either do not consider or have difficulties understanding nicotine strength [11, 45] when choosing VPs, and youth who use VPs often report having never heard of nicotine salts [5]. As perceived addiction to vaping is often mentioned as a key reason driving youth to attempt stop vaping [46], this suggests a potential lack of knowledge among young people regarding nicotine and its role in addiction. Hence, beyond the mandatory health warning, our sampled VPs and their packaging across England, Canada and the United States lack consistent information regarding nicotine levels, composition and how these characteristics affect product addictiveness.

Limitations should be considered when interpreting these findings. The VPs sampled in this study may not entirely reflect the most‐used VPs across the three countries. Popular VPs were sampled within the three countries based on nationally representative data from four online surveys and prominence in shops and online retail platforms, however, the VP market is rapidly evolving and sales data was not used. Pod‐based VPs and refillable devices were not included. Further, selecting flavours based on popular flavour groups as reported in surveys may have overlooked flavours that do not fit into these groups. Some codes (e.g. sensory claims) were partly subjective; this was mitigated by assessment from three independent coders. Finally, we did not conduct statistical analyses because of small sample sizes, therefore, additional studies with larger samples are required. Despite these limitations, this was the first study that sampled and compared packaging of disposable vaping devices and e‐liquids sold in England, Canada and the United States. This study used a thorough coding procedure, with each code checked by three researchers, and provided a comprehensive overview of how VPs are marketed across jurisdictions and countries.

## CONCLUSIONS

In our examination of 48 disposable vapes and 60 e‐liquids, across England, Canada and the United States, we observed variable compliance with local regulations and notable inconsistencies in labelling. Although most vaping products adhered with country‐specific regulations concerning the inclusion of health warnings and product characteristics, compliance was insufficient for less definite requirements related to flavours or marketing elements. Many products featured appeals to flavour‐related sensations, eye‐catching colours and design themes, which may impact product appeal to youth. These findings highlight the need for stricter enforcement, particularly regarding the format and wording of health warnings in the United States. Across all three countries, more comprehensive VP packaging regulations are required, specifically ones addressing marketing and branding attributes that may attract youth. As countries such as England are considering introducing new regulations to restrict packaging features of VPs and change the legal status of certain product types like disposables, these findings should be considered to ensure that regulations minimise youth appeal, while still enabling adults who smoke to use these products for smoking cessation.

## AUTHOR CONTRIBUTIONS

**Matilda K. Nottage:** Conceptualization; data curation; investigation; methodology; resources; writing—original draft; writing—review and editing. **Eve V. Taylor:** Conceptualization; writing—review and editing. **Katherine A. East:** Conceptualization; writing—review and editing. **Ann McNeill:** Conceptualization; data curation; funding acquisition; investigation; methodology; resources; supervision; writing—review and editing. **James F. Thrasher:** Methodology; resources; writing—review and editing. **Jessica L. Reid:** Methodology; resources; writing—review and editing. **David Hammond:** Methodology; resources; writing—review and editing. **Erikas Simonavičius:** Data curation; investigation; methodology; resources; supervision; writing—original draft; writing—review and editing.

## DECLARATION OF INTERESTS

None.

## Supporting information


**Figure S1.** Prominently featured colours across vaping products and their packaging by product type (disposable devices and e‐liquid bottles) and by flavour group.
**Table S1.** Codebook.
**Table S2.** Full product list.
**Table S3.** Product colour data by country and flavour group.

## Data Availability

Data available on request from the authors
