# Supplementary material for: Packaging of disposable vaping products and e‐liquids in England, Canada and the United States: A content analysis
Source: Addiction. 2024 Jul 6;120(3):483–95. doi: 10.1111/add.16611 (PMC11813724; doi:10.1111/add.16611)
Supplement: Supplementary file 1 — Figure S1. Prominently featured colours across vaping products and their packaging by product type (disposable devices and e‐liquid bottles) and by flavour group. Table S1. Codebook. Table S2. Full product list. Table S3. Product colour data by country and flavour group. [file ADD-120-483-s001.docx]

*Packaging of disposable vaping products and e-liquids in England, Canada, and the United States: a content analysis*

**Appendix**

**Figure A1**. Prominently featured colours across vaping products and their packaging by product type (disposable devices and e-liquid bottles) and by flavour group.

*Note*. Up to two prominent colours were coded per product and per packaging.

**Table A1**. Codebook.

| **CODES** |
| --- |
| **Brand** - Brand name (string). |
| **Shop** - Product was bought 1 = in a brick-and-mortar shop or 2 = online |
| **ProdType** - Details of the product type: 1 = Disposable device  2 = Refill – Bottle  3 = Refill – Cartridge (‘pod’)  4 = Tank device  5 = Cartridge device – Disposable cartridge  6 = Cartridge device – Reusable cartridge  7 = Cartridge device – Disposable & Reusable |
| **DevName** - Model name, for devices and pods (string). 99 = N/A |
| **PackElements** - Identifying packaging elements present:  1 = Product + External packaging (e.g., box) 2 = Product + Other packaging only (e.g., loose tag, overwrap) 3 = Product + External packaging + Other packaging 4 = Product only |
| **PackElementsTEXT** - If previous = 2 or 3, please specify (string). 99 = N/A |
| **Leaflet** - Did the packaging include leaflet(s)? 0 = No, 1 = Yes |
| **DevCapacity** - Device's e-liquid capacity (ml). 99 = N/A |
| **Puffs** - Disposable devices: number of puffs per device. 99 = N/A or not indicated  ** Edited: code added after protocol publication* |
| **Nicotine** - Liquid-containing products: Does the product contain any nicotine?  0 = No, 1 = Yes, 99 = N/A |
| **NicoStrengthMG** - Liquid-containing products: Nicotine concentration (mg/ml). 99 = N/A |
| **NicoStrengthPC** - Liquid-containing products: Nicotine concentration (%). 99 = N/A |
| **NicoSalts** - Liquid-containing products: Indication of the presence of nicotine salts in the product. 0 = No, 1 = Yes, 99 = N/A |
| **FlavName** - Liquid-containing products: Flavour name (string). 99= N/A |
| **FlavGroup** - Liquid-containing products: Flavour of the e-liquid:  1 = Tobacco  2 = Fruit  3 = Menthol  4 = Sweets  5 = Mixed  6 = Other  99 = N/A |
| **FlavMixed** - If previous = 5, please enter the flavour groups here (e.g. “1,4” for “caramel tobacco”). 99 = N/A |
| **LiquidQuant** - Liquid-containing products: E-liquid quantity (ml). Per bottle/device/cartridge. 99 = N/A |
| **CountryTarget** - Country which the packaging was designed for or purchased in:  1= UK, 2= US, 3= CAN |
| **BatchNumber** - Batch number present. 0 = No, 1 = Yes (pack), 2 = Yes (product), 3 = Yes (both) |
| **CanadaWarn** Canada: Presence of the compulsory TVPA warning on packaging ("*WARNING: Nicotine is highly addictive.*")? 0 = No, 1 = Yes (pack), 2 = Yes (product), 3 = Yes (both)  **EnglandWarn**  England: Presence of the compulsory TRPR warning on packaging ("*This product contains nicotine which is a highly addictive substance*")? 0 = No, 1 = Yes (pack), 2 = Yes (product), 3 = Yes (both)  **USAWarn**  USA: Presence of the compulsory FDA warning on packaging ("*WARNING: This product contains nicotine. Nicotine is an addictive chemical.*")? 0 = No, 1 = Yes (pack), 2 = Yes (product), 3 = Yes (both) |
| **CanadaWarnSize** Canada: Is the size of the compulsory warning compliant with regulations? (minimum 35% of main display panel, or 2mm type height if the main display panel is <45cm2)  0=No, 1= Yes, 99= N/A  ** Edited: specified ‘type’ height*  **EnglandWarnSize**  England: Is the size of the compulsory warning compliant with regulations? UK: minimum 30% of the front and back panels. 0=No, 1= Yes, 99= N/A  **USAWarnSize**  USA: Is the size of the compulsory warning compliant with regulations? US: minimum 30% of the front and back panels. 0=No, 1= Yes, 99= N/A |
| **NicotineWarnClaim** - Devices: Is the nicotine warning accompanied by a disclaimer saying it only applies when used with a nicotine containing liquid? 0=No, 1=Yes, 99= N/A  ** Edited: specified ‘devices’* |
| **NicoWarnFormat** - Is the format/presentation of the compulsory nicotine warning non-compliant with regulations? (see Guide for country-specific formatting rules)  0 = No, 1 = Yes, 99 = N/A |
| **NicoWarnFormatTEXT** - If previous = 1, please specify (string). |
| **BottleWarn -** E-liquid bottles only: Is the compulsory nicotine warning present on the bottle? 0 = No, 1 = Yes, 99 = N/A |
| **HealthBenefits** - Claims about the product's health benefits, like medicinal properties or effects on energy. 0 = No, 1 = Yes (text), 2 = Yes (image), 3 = Yes (text and image), 99 = N/A |
| **HealthBenefitsTEXT** - If present, please specify claim (string). 99 = N/A |
| **HealthRelative** - Claims about the product being safer than combustible tobacco products.  0 = No, 1 = Yes (text), 2 = Yes (image), 3 = Yes (text and image), 99 = N/A |
| **HealthRelativeTEXT** - If present, please specify claim (string). 99 = N/A |
| **HealthOther** - Claims about e-cigarettes' impact on health, other than health benefits or relative risk compared to tobacco. 0 = No, 1 = Yes (text), 2 = Yes (image), 3 = Yes (text and image), 99 = N/A |
| **HealthOtherTEXT** - If present, please specify claim (string). 99 = N/A |
| **Cessation** - Claims about e-cigarettes' use as a smoking cessation aid.  0 = No, 1 = Yes (text), 2 = Yes (image), 3 = Yes (text and image), 99 = N/A |
| **CessationTEXT** - If present, please specify claim (string). 99 = N/A |
| **QualityMarkings** - Standard quality control markings, such as "CE" (see Guide).  0 = No, 1 = Yes (one), 2 = Yes (several) |
| **QualityClaim** - Claims about the quality of the product.  0 = No, 1 = Yes (text), 2 = Yes (image), 3 = Yes (text and image), 99 = N/A |
| **QualityClaimTEXT** - If present, please specify claim (string). 99 = N/A |
| **OriginClaim** - Claims about where the product was designed and/or manufactured (other than factual address of the manufacturer. E.g., "Made in California").  0 = No, 1 = Yes (text), 2 = Yes (image), 3 = Yes (text and image), 99 = N/A |
| **OriginClaimTEXT** - If present, please specify claim (string). 99 = N/A |
| **PriceIndication** - Is the price indicated on the packaging or product?  0 = No, 1 = Yes (pack), 2 = Yes (product), 3 = Yes (both) |
| **Purchase price** - If previous = 1, 2, or 3, was the purchase price:  0 = same as indicated, 1 = higher than indic., 2 = lower than indic, 99 = N/A |
| **Promo1** - Presence of offers, discounts, or any form of financial promotion on the product or packaging. 0 = No, 1 = Yes (pack), 2 = Yes (product), 3 = Yes (both) |
| **Promo2** - If a promotion was present, was it: 1 = Text, 2 = Image, 3 = Text and image, 99 = N/A |
| **PromoTEXT** - If present, please specify claim (string). 99 = N/A |
| **CostClaims** - Claims about the product being cost-efficient / cheaper than combustible cigarettes. 0 = No, 1 = Yes (text), 2 = Yes (image), 3 = Yes (text and image) |
| **CostClaimsTEXT** - If present, please specify claim (string). 99 = N/A |
| **Recycling** - Indications of recyclability of the product or packaging, or instructions for disposal (factual, eg. logo or brief text).  0 = No, 1 = Yes (text), 2 = Yes (image), 3 = Yes (text and image), 99 = N/A |
| **RecyclingTEXT** - If present, please specify claim (string). 99 = N/A |
| **Sustainability** - Claims about the product being respectful of the environment, eco-friendly, or similar (other than factual indications about recyclability).  0 = No, 1 = Yes (text), 2 = Yes (image), 3 = Yes (text and image), 99 = N/A |
| **SustainabilityTEXT** - If present, please specify claim (string). 99 = N/A |
| **Natural** - For liquid-containing products, is there a reference to ingredients being "natural", "organic", or similar?  0 = No, 1 = Yes (text), 2 = Yes (image), 3 = Yes (text and image), 99 = N/A |
| **NaturalTEXT** - If present, please specify claim (string). 99 = N/A |
| **SocialBenefits** - Claims about the product providing the user with social benefits such as increased ability to socialise or form friendships.  0 = No, 1 = Yes (text), 2 = Yes (image), 3 = Yes (text and image), 99 = N/A |
| **SocialBenefitsTEXT** - If present, please specify claim (string). 99 = N/A |
| **RomanticBenefits** - Claims referring to increased romantic involvement or sexual benefits.  0 = No, 1 = Yes (text), 2 = Yes (image), 3 = Yes (text and image), 99 = N/A |
| **RomanticBenefitsTEXT** - If present, please specify claim (string). 99 = N/A |
| **Endorsements** - Claims that the product or brand is endorsed by an influential person or organisation (eg. celebrity endorsement, designer brand collab).  0 = No, 1 = Yes (text), 2 = Yes (image), 3 = Yes (text and image), 99 = N/A |
| **EndorsementsTEXT** - If present, please specify claim (string). 99 = N/A |
| **Sensory** - Claims relating to sensory perceptions associated with use of the product (e.g., decriptions of flavours or sensations, evocative images like smoke or food)  0 = No, 1 = Yes (text), 2 = Yes (image), 3 = Yes (text and image), 99 = N/A |
| **SensoryTEXT** - If present, please specify claim (string). 99 = N/A |
| **Identity** - Claims relating identity or self-image to the use/purchase of the product (e.g., referring to users as "vapers", claiming a product is for adventurers, travellers, etc.)  0 = No, 1 = Yes (text), 2 = Yes (image), 3 = Yes (text and image), 99 = N/A |
| **IdentityTEXT** - If present, please specify claim (string). 99 = N/A |
| **QRCode** - Presence of QR code(s). 0 = No, 1 = Yes (pack), 2 = Yes (product), 3 = Yes (both) |
| **QRCodeTEXT** - If present, please specify where the code led to. 99 = N/A |
| **Website** - Reference to the manufacturer's website. 0 = No, 1 = Yes (pack), 2 = Yes (product), 3 = Yes (both) |
| **SocialMedia** - Reference to social media platforms.  0 = No, 1 = Yes (pack), 2 = Yes (product), 3 = Yes (both) |
| **SocialMediaTEXT** - If SocialMedia1 = 1, please specify which (string). |
| **MainPackClr-White** - Is this colour used prominently on the packaging?  White or near-white. 0 = No, 1 = Yes, 99 = N/A |
| **MainPackClr-Black** - Prominent black or near-black. 0 = No, 1 = Yes, 99 = N/A |
| **MainPackClr-Metallic** - Prominent metallic (gold, silver). 0 = No, 1 = Yes, 99 = N/A |
| **MainPackClr-Grey** - Prominent grey. 0 = No, 1 = Yes, 99 = N/A |
| **MainPackClr-Brown** - Prominent brown. 0 = No, 1 = Yes, 99 = N/A |
| **MainPackClr-Red** - Prominent red. 0 = No, 1 = Yes, 99 = N/A |
| **MainPackClr-Orange** - Prominent orange. 0 = No, 1 = Yes, 99 = N/A |
| **MainPackClr-Yellow** - Prominent yellow. 0 = No, 1 = Yes, 99 = N/A |
| **MainPackClr-Green** - Prominent green. 0 = No, 1 = Yes, 99 = N/A |
| **MainPackClr-Blue** - Prominent blue. 0 = No, 1 = Yes, 99 = N/A |
| **MainPackClr-Purple** - Prominent purple. 0 = No, 1 = Yes, 99 = N/A |
| **MainPackClr-Pink** - Prominent pink. 0 = No, 1 = Yes, 99 = N/A |
| **MainProdClr-White** - Is this colour used prominently on the product (device, pod, or bottle)? White or near-white. 0 = No, 1 = Yes, 99 = N/A |
| **MainProdClr-Black** - Prominent black or near-black. 0 = No, 1 = Yes, 99 = N/A |
| **ProminentProductColour-Metallic** - Prominent metallic. 0 = No, 1 = Yes, 99 = N/A |
| **MainProdClr-Grey** - Prominent grey. 0 = No, 1 = Yes, 99 = N/A |
| **MainProdClr-Brown** - Prominent brown. 0 = No, 1 = Yes, 99 = N/A |
| **MainProdClr-Red** - Prominent red. 0 = No, 1 = Yes, 99 = N/A |
| **MainProdClr-Orange** - Prominent orange. 0 = No, 1 = Yes, 99 = N/A |
| **MainProdClr-Yellow** - Prominent yellow. 0 = No, 1 = Yes, 99 = N/A |
| **MainProdClr-Green** - Prominent green. 0 = No, 1 = Yes, 99 = N/A |
| **MainProdClr-Blue** - Prominent blue. 0 = No, 1 = Yes, 99 = N/A |
| **MainProdClr-Purple** - Prominent purple. 0 = No, 1 = Yes, 99 = N/A |
| **MainProdClr-Pink** - Prominent pink. 0 = No, 1 = Yes, 99 = N/A |
| **BottleShape** - The shape of the bottle is: (see Guide) 1 = Common 2 = Uncommon (body and/or lid) 99 = N/A |
| **BottleShapeTEXT** - If previous = 2, specify (string). |
| **DeviceShape** - The device's shape is (see Guide):  1 = Rectangular, 2 = Curved/Cylindrical, 3 = Mixed, 99 = N/A |
| **OtherShapeSize** - Is there any other notable element related to shape or size of the product or package? 0 = No 1 = Yes |
| **OtherShapeSizeTEXT** - If previous = 1, please justify (string). |
| **BrandFont** - The font used for the brand name (as depicted on the packaging/product - see Guide) is: 0 = Non-stylised, 1 = Lightly stylised, 2 = Heavily stylised, 99 = N/A |
| **BrandLogoPlace** - The brand logo and/or brand name is present on: 1 = Packaging 2 = Product 3 = Both |
| **BrandLogo** - Presence of a brand logo (not just the brand name/font, but any graphic element accompanying it). 0 = No, 1 = Yes (pack), 2 = Yes (product), 3 = Yes (both), 99 = N/A |
| **LogoDetails** - If present, the brand logo represents (see Guide):  1 = Something abstract or geometric  2 = A cartoon character, animal, or mascot  3 = A natural element (e.g., leaf, cloud, sun)  4 = Other  99 = N/A |
| **LogoDetailsTEXT** - Please specify logo details if applicable (string). 99 = N/A |
| **ProductImage** - Is there an image representing the product (device, cartridge, or bottle) on the external packaging? 0 = No, 1 = Yes, 99 = N/A |
| **FlavourImage** - For liquid-containing products: is there a flavour-related photo or illustration on the packaging or product?  0 = No, 1 = Yes (pack), 2 = Yes (product), 3 = Yes (both), 99 = N/A |
| **ProdGraph1** - Is there any other graphic/illustrative element on the product?  0 = No, 1 = Yes, 99 = N/A |
| **ProdGraph2** - The graphic/illustrative element is:  1 = Abstract (e.g., pattern, glitter)  2 = Figurative (e.g., character, natural element, object)  3 = Both 99 = N/A  ** Edited: added option 3* |
| **ProdGraphTEXT** - Please specify (string). 99 = N/A |
| **PackGraph1** - Is there any other graphic/illustrative element on the packaging?  0 = No, 1 = Yes, 99 = N/A |
| **PackGraph2** - The graphic/illustrative element is:  1 = Abstract (e.g., pattern, glitter)  2 = Figurative (e.g., character, natural element, object)  3 = Both 99 = N/A  ** Edited: added option 3* |
| **PackGraphTEXT** - Please specify (string). 99 = N/A |
| **YouthGenre** - Does the product or packaging feature elements of youth-associated genres, such as humour, magic, fantasy, violence, or sports/adventure? (*from CAY index, Padon et al, 2018*)  0 = No, 1 = Yes, 2 = Unsure |
| **YouthGenreTEXT** - Please justify (string). |

**Table A2.** Full product list

| **Country** | **Type** | **Name** |
| --- | --- | --- |
| Canada | Disposable | Boosted Bar Plus - Blackcurrant Lychee (20mg) |
|  |  | Elfbar - Sour Cdy (20mg) |
|  |  | Flavour Beast - Mad Mango Peach (20mg) |
|  |  | Genie Air Slender - Cool Mint (20mg) |
|  |  | Vice - Tobacco (20mg) |
|  |  | Allo Ultra 1600 Disposable - Frost (20mg) |
|  |  | Allo Ultra 800 Disposable - Classic Tobacco (20mg) |
|  |  | Envi Apex Disposable - Green Apple (20mg) |
|  |  | Envi Boost Disposable - Cocoa (20mg) |
|  |  | Envi Boost Disposable - Fresh Mint (20mg) |
|  |  | Ghost Max Disposable - Smooth Tobacco (20mg) |
|  |  | Ghost Box Disposable Cherry Grape (20mg) |
|  |  | Ghost Max Disposable - Roots (20mg) |
|  |  | Vuse Go Disposable - Berry Blend (20mg) |
|  |  | Vuse Go Disposable - Creamy Tobacco (20mg) |
|  |  | Vuse Go Disposable - Mint Ice (20mg) |
|  | Refill bottle | Indulge - Nutty (6mg) |
|  |  | Koil Killaz - Roundhouse (12mg) |
|  |  | Lix - Mint Condition (3mg) |
|  |  | (Apple) Drop - Berries (6mg) |
|  |  | Boosted - Mint (3mg) |
|  |  | Lift - Golden Tobacco (12mg) |
|  |  | E Fizz - Twister Olly (0mg) |
|  |  | Iced Up - Mint Ice (0mg) |
|  |  | (Lemon) Drop – Mango (0mg) |
|  |  | Vice Vapour - Mango Passionfruit Guava (0mg) |
|  |  | Don Cristo - Custard Cigar (0mg) |
|  |  | Shipwreck Kraken - Fresh Tobacco (0mg) |
|  |  | 1Hundred - Apple Melon (20mg) |
|  |  | Allday Vapor - El Bacco (20mg) |
|  |  | Crave - Hazel (20mg) |
|  |  | Canada E Clouds – Slayer Salt (20mg)  ** ‘Dragon Slayer Salt’ on retailer website when purchased, labelled ‘Slayer Salt’ when received* |
|  |  | Dinner Lady – Original Blackberry (3mg)  ** ‘Blackberry crumble’ on retailer website when purchased, labelled ‘Original blackberry’ when received* |
|  |  | Vital - Mango Dragonfruit (0mg) |
|  |  | Naked - Arctic Air (20mg) |
|  |  | Banana Bang - Strawberry Orange (20mg) |
| USA | Disposable | SWFT LUX 3500 - Kiwi Berry (50mg) |
|  |  | SWFT Mod 5000 - Bubblegum (50mg) |
|  |  | SWFT Mod 5000 – Peppermint (50mg) |
|  |  | Hyde Retro Rave 5000 - Loops (50mg) |
|  |  | Hyde Nbar Mini 2500 - Sour Apple Ice (50mg) |
|  |  | Hyde Edge 1500 - Raspberry Watermelon (50mg) |
|  |  | Flum Pebble 6000 - Menthol (50mg) |
|  |  | Flum Neno 600 - Apple Ice (50mg) |
|  |  | Flum Gio 3000 - Tobacco Cream (50mg) |
|  |  | Daze Ohmlet 7000 - Blueberry Peach (50mg) |
|  |  | Daze Egge 3000 - Cake Pop (50mg) |
|  |  | Daze Egge 3000 - 7obbaco (50mg) |
|  |  | ElfBar BC5000 - Mango Peach (50mg) |
|  |  | AirBar Diamond - Watermelon Apple Ice (50mg) |
|  |  | PodMesh 5500 - Jewel Tobacco (55mg) |
|  |  | EscoBars - Spearmint (50mg) |
|  |  | EscoBars - GummyBear (50mg) |
|  | Refill bottle | Candy King - Batch (50mg) |
|  |  | Keep it 100 - Tropical Blast (0mg) |
|  |  | Coastal Clouds - Blueberry Limeade (0mg) |
|  |  | (Fruit) Monster - Passionfruit Orange Guava (24mg) |
|  |  | (Tobacco) Monster - Smooth (40mg) |
|  |  | The Cloud Chemist - Cereal Science (3mg) |
|  |  | Halo - Subzero (0mg) |
|  |  | Twist - Mint No1 (35mg) |
|  |  | Cloud Nurdz - Peach Blue Raspberry (3mg) |
|  |  | InneVape – Carousel (0mg) |
|  |  | Air Factory - Mint (3mg) |
|  |  | Four Seasons - Turkish Tobacco (18mg) |
|  |  | Fruitia - Blood Orange Cactus (3mg) |
|  |  | SVRF - Refreshing (3mg) |
|  |  | Juice Head - Peach Pear (0mg) |
|  |  | Naked 100 - Crisp Menthol (0mg) |
|  |  | Naked 100 - Tobacco (0mg) |
|  |  | Glas Basix Salt - PBLS (30mg) |
|  |  | Juice Man - Unicorn Frappe Salts (50mg) |
|  |  | Mad Hatter - Classic Tobacco Salt (25mg) |
| England | Disposables | Geek Bar S600 - Strawberry (20mg) |
|  |  | Geek Bar E600 - Unicorn Milkshake (20mg) |
|  |  | Geek Bar E600 - Fresh Mint (20mg) |
|  |  | Geek Bar 575 - Tobacco (20mg) |
|  |  | Flum Float - Fruity Hawaii (20mg) |
|  |  | Elux Bar 600 - Pear Kiwi Lemonade (20mg) |
|  |  | HQD - Sky Mint 1200 (20mg) |
|  |  | Solo+ 600 - Cherry Cola (20mg) |
|  |  | ElfBar Lost Mary BM600 - Menthol (20mg) |
|  |  | ElfBar NC600 - Blackcurrant (20mg) |
|  |  | ElfBar T600 - Strawberry Cheesecake (20mg) |
|  |  | ElfBar 600 - Cream Tobacco (20mg) |
|  |  | Magic Bar 600 - Tobacco (20mg) |
|  |  | Smok Mbar - Blueberry Soda (20mg) |
|  |  | Smok Novobar - Peach Ice (20mg) |
|  | Refill bottles | Milkman - Apple Pie 50ml (0mg) |
|  |  | Pocket Fuel - Menthol Mist 50ml (0mg) |
|  |  | Vampire Vape - Smooth Tobacco 550ml (0mg) |
|  |  | Yeti - Grape 100ml (0mg) |
|  |  | Aisu - Mango 50ml (+nicotine shot) (18mg) |
|  |  | Riot Salt - Blue Burst (10mg) |
|  |  | Pod Salts Nexus - Strawberry Watermelon Kiwi (10mg) |
|  |  | Nasty Salt - Menthol (20mg) |
|  |  | Just Juice - Tobacco Club Vanilla Toffee (11mg) |
|  |  | Dinner Lady - Caramel Tobacco Salt (20mg) |
|  |  | Vapouriz - Classic Tobacco (0mg) |
|  |  | V4V4pour - Watermelon 10ml (0mg) |
|  |  | Simple Essentials - Vanilla Custard (0mg) |
|  |  | VIP - British Gold (3mg) |
|  |  | Edge - Very Menthol (6mg) |
|  |  | 88 Vape - Raspberry Ripple (11mg) |
|  |  | Element - Fresh Squeeze (6mg) |
|  |  | Doozy Vape - Nektar (6mg) |
|  |  | El Diablo - Beelzebub (0mg) |
|  |  | Atom - Menthol (0mg) |

**Table A3.** Product colour data by country and flavour group

|  | External packaging (*n*=75) | | | | | | | | | | | | Products (*n*=108) | | | | | | | | | | | |
| --- | --- | --- | --- | --- | --- | --- | --- | --- | --- | --- | --- | --- | --- | --- | --- | --- | --- | --- | --- | --- | --- | --- | --- | --- |
|  | **Disposables** | | | | | | **Bottles** | | | | | | **Disposables** | | | | | | **Bottles** | | | | | |
|  | Tob | Fru | Men | Swe | Mix | **All** | Tob | Fru | Men | Swe | Mix | **All** | Tob | Fru | Men | Swe | Mix | **All** | Tob | Fru | Men | Swe | Mix | **All** |
| **England** | | | | | | | | | | | | | | | | | | | | | | | | |
| White | 1 | 1 | 0 | 0 | 3 | 5 | 0 | 1 | 1 | 1 | 2 | 5 | 0 | 1 | 0 | 0 | 0 | 1 | 0 | 4 | 1 | 1 | 3 | 9 |
| Black | 0 | 1 | 0 | 0 | 0 | 1 | 0 | 0 | 0 | 0 | 1 | 1 | 0 | 0 | 0 | 0 | 1 | 1 | 3 | 3 | 0 | 1 | 1 | 8 |
| Neutrals | 1 | 0 | 1 | 0 | 1 | 3 | 2 | 1 | 0 | 0 | 1 | 4 | 2 | 0 | 1 | 0 | 1 | 4 | 1 | 1 | 0 | 0 | 1 | 3 |
| Warm | 0 | 3 | 0 | 2 | 1 | 6 | 0 | 3 | 0 | 0 | 1 | 4 | 0 | 3 | 0 | 1 | 2 | 6 | 0 | 4 | 0 | 0 | 0 | 4 |
| Cool | 0 | 1 | 3 | 0 | 1 | 5 | 0 | 0 | 2 | 0 | 0 | 2 | 0 | 1 | 3 | 0 | 1 | 5 | 0 | 1 | 4 | 0 | 0 | 5 |
| **Canada** | | | | | | | | | | | | | | | | | | | | | | | | |
| White | 1 | 1 | 1 | 0 | 0 | 3 | 0 | 0 | 0 | 0 | 0 | 0 | 0 | 0 | 0 | 0 | 0 | 0 | 1 | 3 | 1 | 2 | 0 | 7 |
| Black | 2 | 2 | 0 | 1 | 0 | 5 | 0 | 0 | 0 | 0 | 0 | 0 | 2 | 2 | 1 | 2 | 0 | 7 | 2 | 2 | 0 | 0 | 0 | 4 |
| Neutrals | 1 | 0 | 0 | 1 | 0 | 2 | 0 | 0 | 0 | 0 | 0 | 0 | 2 | 0 | 0 | 0 | 0 | 2 | 1 | 1 | 0 | 0 | 0 | 2 |
| Warm | 0 | 0 | 0 | 0 | 0 | 0 | 0 | 0 | 0 | 0 | 0 | 0 | 0 | 0 | 0 | 0 | 0 | 0 | 0 | 1 | 0 | 0 | 2 | 3 |
| Cool | 0 | 3 | 3 | 1 | 0 | 7 | 0 | 0 | 0 | 0 | 0 | 0 | 0 | 3 | 3 | 1 | 0 | 7 | 0 | 3 | 3 | 0 | 0 | 6 |
| **USA** | | | | | | | | | | | | | | | | | | | | | | | | |
| White | 2 | 2 | 1 | 2 | 1 | 8 | 0 | 2 | 1 | 1 | 1 | 5 | 1 | 0 | 1 | 0 | 1 | 3 | 1 | 5 | 4 | 2 | 2 | 14 |
| Black | 0 | 0 | 0 | 0 | 2 | 2 | 1 | 2 | 1 | 0 | 1 | 5 | 0 | 0 | 0 | 0 | 2 | 2 | 1 | 4 | 2 | 0 | 1 | 8 |
| Neutrals | 2 | 1 | 0 | 0 | 1 | 4 | 2 | 0 | 0 | 0 | 0 | 2 | 2 | 0 | 0 | 0 | 0 | 2 | 3 | 0 | 0 | 0 | 0 | 3 |
| Warm | 0 | 1 | 1 | 3 | 0 | 5 | 0 | 6 | 1 | 0 | 0 | 7 | 0 | 4 | 1 | 4 | 0 | 9 | 0 | 6 | 0 | 0 | 0 | 6 |
| Cool | 0 | 2 | 2 | 0 | 2 | 6 | 0 | 3 | 2 | 0 | 1 | 6 | 0 | 0 | 2 | 1 | 2 | 5 | 0 | 3 | 4 | 0 | 1 | 8 |
| **x-country** | **10** | **18** | **12** | **10** | **12** | **62** | **5** | **18** | **8** | **2** | **8** | **41** | **9** | **14** | **12** | **9** | **10** | **54** | **13** | **41** | **19** | **6** | **11** | **90** |
| White | 4 | 4 | 2 | 2 | 4 | 16 | 0 | 3 | 2 | 2 | 3 | 10 | 1 | 1 | 1 | 0 | 1 | 4 | 2 | 12 | 6 | 5 | 5 | 30 |
| Black | 2 | 3 | 0 | 1 | 2 | 8 | 1 | 2 | 1 | 0 | 2 | 6 | 2 | 2 | 1 | 2 | 3 | 10 | 6 | 9 | 2 | 1 | 2 | 20 |
| Neutrals | 4 | 1 | 1 | 1 | 2 | 9 | 4 | 1 | 0 | 0 | 1 | 6 | 6 | 0 | 1 | 0 | 1 | 8 | 5 | 2 | 0 | 0 | 1 | 8 |
| Warm | 0 | 4 | 1 | 5 | 1 | 11 | 0 | 9 | 1 | 0 | 1 | 11 | 0 | 7 | 1 | 5 | 2 | 15 | 0 | 11 | 0 | 0 | 2 | 13 |
| Cool | 0 | 6 | 8 | 1 | 3 | 18 | 0 | 3 | 4 | 0 | 1 | 8 | 0 | 4 | 8 | 2 | 3 | 17 | 0 | 7 | 11 | 0 | 1 | 19 |

**Note**. Tob = tobacco, Fru = fruit, Men = menthol, Swe = sweets/desserts, Mix = mixed. One product or pack may feature more than one primary colour. External packaging was not present on all products as detailed in Table 2.
